# Antibiotics Use in Primary Care during COVID-19

**DOI:** 10.3390/antibiotics11060744

**Published:** 2022-05-31

**Authors:** Carl Llor

**Affiliations:** 1 Department of Public Health, General Practice, University of Southern Denmark, 5000 Odense, Denmark; cllor@health.sdu.dk; 2 Via Roma Health Centre, University Institute in Primary Care Research Jordi Gol i Gurina, 08007 Barcelona, Spain

During national health emergencies such as the COVID-19 pandemic, a robust primary care system plays a crucial role in triaging, educating patients and testing. Notwithstanding, the primary care system worldwide has been underprepared for the pandemic, largely because it had been weakened by chronic underinvestment and workforce shortages. In turn, the primary care system experienced massive setbacks from the pandemic, with significant reductions in visit volume as patients stopped seeking in-person care, important organizational challenges with more phone consultations and many countries adopted digital-first strategies, remote monitoring and telehealth platforms to enable healthcare provision without physical interactions [1].

Due to the reduction in face-to-face consultations, an unprecedented decrease in community antibiotic consumption has been observed in many countries between 2019 and 2020 [2]. This Special Issue includes several research articles that discuss how antibiotic consumption and prescribing has been altered due to the COVID-19 pandemic and how doctors have experienced this changing organizational system in primary care. In a qualitative study, Borek et al. [3] found that the threshold for prescribing antibiotics for respiratory tract infections among English primary care doctors decreased during the first few months of the pandemic, caused by the change to remote consultations and the high uncertainty about the role of antibiotics for this infection that was present at the beginning of the pandemic, resembling the high usage of antibiotics given in the hospital setting for treating community-acquired pneumonia. As the epidemic continued and professionals gained more experience and evidence emerged about the lack of antibacterial treatment for COVID-19, antibiotic therapy was again reserved for patients with signs of laboratory-confirmed bacterial infections and the threshold to prescribe antibiotics for respiratory tract infections returned to the pre-pandemic levels. Primary care physicians perceived that the COVID-19 pandemic had little impact on antibiotic prescribing rates for infections, such as urinary and skin infections, with the exception of respiratory tract infections.

Despite lowering the threshold to prescribe antibiotics for respiratory infections during the first wave of the COVID-19 pandemic, the lack of face-to-face consultations, mainly during the first wave of the pandemic, resulted in a lower net antibiotic prescribing in primary care. In a Dutch study, Boeijen et al. [4] found that the total number of infectious diseases declined as soon as the pandemic started, in particular gastrointestinal tract infections and urinary tract infections, compared to the prevalence observed in 2019. This resulted in a 34% reduction in the number of antibiotics prescribed during the first year of the pandemic. Importantly, this reduction in the percentage of the antibiotics prescribed achieved in 2020 did not lead to an increase in the number of complications from respiratory tract infections. Conversely, the number of cases of pneumonia and mastoiditis among Dutch individuals even declined during the first year of the pandemic compared to the same population before the pandemic.

A French study compared the prescribing rates of antibiotics in 2019 and 2020 among primary care doctors, pediatricians and dentists [5]. In comparison with 2019, the prescription rates decreased in 2020, mainly among pediatricians, whereas the reduction was lower among general practitioners, with a slight reduction among elderly individuals and those with comorbidities. As mentioned by the authors of this article, this significant reduction in antibiotics prescribed for children, approximately 35%, could have been explained by the promotion of preventive measures against the COVID-19 pandemic, resulting in a reduction in viral infections in general. However, the authors found an increase in the number of antibiotics prescribed in 2020 among dentists, with ample use of broad-spectrum antibacterial agents. This point is crucial as strategies to tackle the antibiotic prescribing inappropriateness targeting dentists are lacking.

Barbieri et al. [6] observed even higher antibiotic prescribing reductions among Italian children compared to the previous paper, with an 80% reduction in antibiotic prescribing rates for children with respiratory tract infections compared to the pre-pandemic period. However, due to uncertainty, pediatricians tended to use more broad-spectrum antibiotics during the first few months of the pandemic, mainly for upper airway infections, such as amoxicillin and clavulanate, at the detriment of first-line antibiotics.

Finally, with the use of a large Catalonian primary care database which included all the suspected and confirmed COVID-19 infections during the first four months of the pandemic, a composite severity endpoint, comprising pneumonia, hospital admission and death due to COVID-19, was found to be more frequent among individuals who has previously consumed antibiotics compared to those who did not. This correlation was stronger for high antibiotic exposure, exposure to the highest priority critically important antimicrobials and recent exposure, after potential confounders were adjusted by logistic regression [7]. This significant correlation between previous antibiotic exposure and increased severity of the COVID-19 disease is the result of the emerging evidence that antibiotic exposure alters the microbiome by reducing the number of species, and this is believed to impair antiviral immunity.

This Special Issue collects multidisciplinary research papers focusing on how antibiotic prescribing has changed due to the COVID-19 pandemic. The different contributions collected in this issue constitute valuable knowledge for primary care doctors and researchers working in the field of the rational use of antibiotics during a pandemic. All these research articles highlight the safety of withholding antibiotic therapy for common infections in primary care. As we have witnessed with COVID-19, everyone is responsible for implementing and developing actions to control or stop the spread of disease, but it is equally important to change dangerous practices through the appropriate and prudent use of antibacterial agents [8]. The COVID-19 pandemic has given us the opportunity to identify mechanisms for more prudent antibiotic use and how to combat antimicrobial resistance in general.
